# Beyond appearance: how clothing shapes self-confidence through self-expression and social identity in a collectivist context

**DOI:** 10.3389/fpsyg.2026.1933506

**Published:** 2026-10-13

**Authors:** Md Raduan, Md Merajur Rahman, A. K. M. Ashiqur Rahman Mazumdar

**Affiliations:** 1 Pabna Textile Engineering College, Pabna, Bangladesh; 2 Iowa State University, Ames, IA, United States; 3 Bangladesh University of Textiles, Dhaka, Bangladesh

**Keywords:** Bangladesh, clothing-related confidence, collectivist culture, enclothed cognition, impression management, self-confidence, self-construal, social identity

## Abstract

Using a sequential explanatory mixed-methods design, this study examined how clothing relates to self-confidence among 312 Bangladeshi university students (72. 4% male), testing whether the internal and external pathways described by enclothed cognition and impression management theory remain separable when the self is culturally construed as interdependent rather than independent. Exploratory and confirmatory factor analyses supported a provisional, internally cross-validated two-factor structure distinguishing Self-Expression and Performance Confidence from Social Identity and External Influence Confidence, though the two factors were strongly correlated (*r* = 0.79), and a second-order model fit the data equivalently well. Female students reported higher self-expression confidence than male students [t (310) = −3.48, p =0.001], and hierarchical regression showed that psychological predictors, social influence confidence and appearance-related academic-performance belief, explained substantial additional variance beyond demographics (ΔR^2^ = 0.356), together accounting for 39.4% of variance in self-expression confidence. Thematic analysis of 22 interviews and 183 open-ended responses identified five themes: comfort and authenticity; clothing as context-specific “armor”; the social gaze; identity negotiation; and inner self-reliance, which converged with, complemented, and in one case qualified the quantitative findings. The strong association between internal and external confidence dimensions is consistent with self-construal theory and prior cross-cultural evidence that impression management operates more automatically for people from collectivist backgrounds, suggesting these pathways may be less distinguishable in this context than in the largely Western samples on which the underlying theories were developed. The Social Identity dimension showed weaker psychometric performance and should be interpreted as exploratory. Findings extend enclothed cognition and impression management theory to a collectivist South Asian context and offer directions, rather than validated recommendations, for higher education, student counseling, career preparation, and fashion education.

## Introduction

University students face evaluative moments almost constantly: a class presentation, a viva, a job interview, a first meeting with classmates in a new semester. In these moments, confidence matters, and clothing is one of the tools students reach to manage it. Beyond keeping the body covered, what a person wears carries meaning, and that meaning does not stay silent. It shapes how the wearer feels and how others respond to them (Barnard, 2002, 2020). For many students, then, clothing is not incidental to confidence. It is one of the resources through which confidence gets built, tested, and sometimes lost.

Two theories are usually invoked to explain this. Enclothed cognition holds that clothing carries symbolic meaning, and that putting it on can shift the wearer's own thinking, mood, and behavior (Adam and Galinsky, 2012). Impression management theory approaches the same territory from the outside in: people dress to shape how others see them, and the reactions they get back feed into how they see themselves (Peluchette and Karl, 2007). Read together, these two accounts sketch clothing as a switch that can be flipped from the inside (self-expression, comfort, a felt sense of readiness) or from the outside (how a classmate or interviewer reads the outfit, and what that reading does to interaction). What is less often asked is whether these are really two separate switches, or one switch with two labels.

That question has barely been tested outside individualist, Western settings. Enclothed cognition's founding studies used lab coats; impression management's evidence base leans heavily on Western workplaces and business dress (Adam and Galinsky, 2012; Peluchette and Karl, 2007). Both theories, in other words, were built where the self is typically theorized as independent, bounded, and separable from the social world around it (Markus and Kitayama, 1991). Whether the same internal/external split holds where the self is construed differently is an open question, and it is not a small one. Markus and Kitayama's account of independent and interdependent self-construal argues that in collectivist cultures, the self is not first formed privately and then presented socially. It is relational from the start: social context, belonging, and the expectations of others are part of how a person experiences their own identity, not an external layer added on top of it. If that is right, then in a collectivist setting the boundary enclothed cognition and impression management quietly assume, between what I feel about my clothes and what others think of them, may not hold in the same way. The two pathways these theories describe as distinct might instead run closer together, even blur into each other. This is not purely theoretical speculation. Experimental work on survey responding has found that impression management operates as a relatively automatic process for people from collectivist cultural backgrounds, in contrast with the more effortful, deliberate process typical of individualists (Riemer and Shavitt, 2011), suggesting the internal/external boundary these theories assume may genuinely function differently depending on cultural self-construal.

Bangladesh offers a useful test case, and not only because it is under-researched. Clothing here is rarely a purely individual style choice. A saree or a panjabi carries family expectation, religious and cultural weight, and a sense of belonging that is felt as much as chosen. Prior work on clothing and confidence has tended to lump self-expression, social signaling, and performance beliefs together without much conceptual separation, which makes it hard to say which processes are doing the work (Kwon, 1991; Peluchette and Karl, 2007). And on the methods side, very little South Asian research has paired a quantitative scale with qualitative interviews to ask not just whether clothing shapes confidence, but how students themselves describe that happening.

This study takes up both gaps. It asks how clothing shapes confidence among Bangladeshi university students, using a sequential explanatory mixed-methods design, and in doing so, tries to answer the theoretical question raised above rather than sidestep it. Four things follow from that aim, and they are the direct answer to what this paper is contributing beyond a geographic replication. First, the theoretical gap being filled is the untested assumption, carried over from individualist research settings, that internal and external pathways to clothing confidence operate as separable processes; this study tests that assumption empirically rather than taking it for granted. Second, a collectivist context changes the theory because self-construal theory predicts that interdependent selves do not process social evaluation as an input separate from self-perception, they experience the two as entangled, which generates a specific, falsifiable expectation: internal and external confidence dimensions should show a tighter empirical relationship here than dual-process models built on independent-self samples would predict. Third, this means collectivism is treated here as something that can modify or extend enclothed cognition and impression management, not just a new backdrop for applying them unchanged; the study is designed to find out whether that modification shows up in the data. Fourth, the two-factor structure proposed here, Self-Expression and Performance Confidence alongside Social Identity and External Influence Confidence, is not novel merely because no one has named these dimensions before in a Bangladeshi sample. Its theoretical interest lies in whether the two factors turn out to be as cleanly separable as Western-derived theory implies, or whether their empirical relationship itself becomes evidence for how self-construal reshapes the enclothed cognition/impression management divide.

Accordingly, the study addresses the following research question: *How does clothing influence self-confidence among university students in Bangladesh, and does a collectivist self-construal alter the relationship between internal and external pathways to that confidence?* The specific objectives are to: (a) identify how clothing choices relate to different dimensions of self-confidence; (b) examine how gender and socio-cultural background shape these relationships; and (c) explore students' own accounts of how clothing affects first impressions, social interaction, and perceived performance. By grounding enclothed cognition and impression management in a collectivist, non-Western setting, and by treating their theorized separateness as something to be tested rather than assumed, this study aims to extend both frameworks rather than simply relocate them.

### Internal mechanism: enclothed cognition and self-expression

Enclothed cognition starts from a simple claim: clothing is not just seen, it is worn, and wearing something with symbolic meaning can change how a person thinks and feels (Adam and Galinsky, 2012). This foundational claim has faced scrutiny in recent years. A z-curve and meta-analytic reassessment found that some of the earliest, most widely cited enclothed cognition demonstrations show weaker evidential value than more recent studies, suggesting the effect is real but narrower and more context-dependent than early accounts implied (Horton et al., 2025). This matters for confidence specifically, since it suggests clothing might affect motivation, a sense of readiness, and self-perception before a student ever walks into a classroom or an interview room.

There is a reasonable body of evidence behind this. Young adults use clothing to signal identity to themselves as much as to others (Kwon, 1991; McNeill, 2018), and in academic or professional settings, formal or well-put-together attire tends to get linked with seriousness and competence in the wearer's own mind, not only in observers' judgments (Peluchette et al., 2006; Peluchette and Karl, 2007). Clothing has also been tied to psychological wellbeing more broadly, through embodiment, mood, and identity work (Masuch and Hefferon, 2014), and Johnson et al. (2014) bring together a wide range of findings showing that clothing consistently touches both self-perception and how others perceive the wearer.

A related strand looks at fashion involvement, body image, and self-objectification (Blaso et al., 2025; Hollett and Challis, 2023; Hollett et al., 2024). Fashion gives people a language for talking about their bodies, and being more invested in it tends to raise body awareness, for better or worse, which in turn touches confidence and mood. Clothing choices track with body image and personality more generally (Jung et al., 2001; Rudd and Lennon, 2000; Sontag and Lee, 2004; Tiggemann and Lacey, 2009), and Entwistle (2023) captures this well in calling dress an “embodied social practice,” sitting right at the point where individual identity meets cultural expectation.

Still, this literature has a blind spot. It was built almost entirely in individualist settings, on garments like lab coats, uniforms, and suits (Adam and Galinsky, 2012), and it tends to study the internal side of clothing in isolation, as though what happens in a person's head about their outfit is untouched by what is happening socially around them. That separation is itself a theoretical choice, one that follows naturally from an independent view of the self (Markus and Kitayama, 1991), and it is precisely the choice this study wants to test rather than inherit. Where dress carries deep ties to modesty, tradition, and belonging, as it does in Bangladesh (Rahman et al., 2021), the internal experience of getting dressed may already contain the social meaning that Western enclothed cognition research treats as a separate, later step.

### External mechanism: impression management and social identity

The other half of the picture is social. Impression management theory says people dress with an eye to how they will be read, because clothing is a visible signal of identity, competence, and status (Peluchette and Karl, 2007; Hester and Hehman, 2023), and the feedback that signal generates, spoken or not, feeds back into self-confidence.

This shows up clearly in academic and professional life. Attire shapes hiring judgments and workplace evaluation (Furnham et al., 2013), and professional dress often works as what Rafaeli et al. (1997) call an “informative role symbol,” letting someone step into a particular identity just by putting on the right clothes. In healthcare and professional communication settings, formal attire raises perceived trustworthiness and competence (Dayani et al., 2022; Maruani et al., 2013), and even perceived charisma in public speaking tracks with what a speaker wears (Brem and Niebuhr, 2021).

Clothing does more than manage impressions, though. It offers comfort and a sense of security (Kwon and Shim, 1999; Kang et al., 2013; Koksal, 2014; Moody et al., 2010; McNeill and Venter, 2019), and confidence built through personal style seems to hold up best when that style feels authentic rather than performed (Roster, 2024). Rubinstein (2018) frames clothing as a semiotic system, carrying authority and belonging the way language does, which fits with older accounts of self-presentation and image management (Rafaeli and Pratt, 1993; Goffman, 2023). In Bangladesh specifically, younger consumers' fashion choices already show this mix of internal preference and external, sustainability-linked values pulling on the same decision (Minhus and Huie, 2022).

Even so, most of this research looks at the observer's side of the exchange, how others judge the wearer, rather than how the wearer absorbs and internalizes that judgment. And like enclothed cognition, it is mostly Western in origin. In a collectivist setting, dress answers not just to personal taste but to community expectation and social legitimacy, which raises a real possibility: that the internal and external dimensions here are not two processes running in parallel, but one process viewed from two angles. Self-construal theory gives a reason to expect exactly this. If the interdependent self-experiences social belonging as constitutive of identity rather than external to it (Markus and Kitayama, 1991), then impression management is not simply feeding information into a separate internal system. It may be part of the same system.

### Cultural context: collectivism, traditional attire, and gender

Culture and gender shape what clothing means are often more than the clothing itself. In Bangladesh, garments like the saree and the panjabi carry weight well beyond aesthetics: pride, belonging, religious and cultural continuity (Mahamud, 2016). Sarees move across casual and formal occasions alike, valued for both look and meaning, while the panjabi is closely tied to religious and cultural festivals, reinforcing a shared identity each time it is worn (Kaikobad et al., 2014; Minhus and Huie, 2021). Festivals and heritage events give these garments further visibility (Minhus et al., 2022), and their colors and patterns reflect regional history built up over generations (Tajuddin, 2018).

Cross-cultural work backs up the idea that clothing's psychological effects are not fixed. (Hannover and Kühnen 2002) found that formal dress shifted how formally people described themselves, and Rucker et al. (1999) showed that ethnic minority groups can read the same symbolic clothing differently depending on their social position and prospects. Clothing, in short, does not mean the same thing everywhere, because the self-reading is not built the same way everywhere either. This is the core claim behind self-construal theory (Markus and Kitayama, 1991): independent selves in individualist cultures draw a firmer line between “who I am” and “how others see me,” while interdependent selves in collectivist cultures experience those as more continuous. This is not a claim self-construal theory makes alone. Symbolic interactionist accounts of identity reach a similar place from a different direction, treating the self as something built through ongoing social interaction rather than something that exists first and gets expressed afterward (Ambás and Gárgoles, 2025). If clothing speaks to the self, and the self is constructed differently across cultures, then clothing's psychological effects should differ in structure, not just in strength. That is a testable claim, and it is the one this study is built around.

Gender adds another layer, Kwon (1994) found that men more often link appropriate dress to competence and trustworthiness, while women's clothing choices tend to connect more directly to body image and identity, and women frequently report feeling more confident when their clothing matches how they want to be seen, both by themselves and others (Stolovy, 2021). This fits broader work treating clothing as a stage on which gender norms get performed, negotiated, or pushed back against (Crane, 2000; Garber, 2012). Clothing also works as an identity signal for groups outside the mainstream. Historically, LGBTQ+ communities have used dress cues to find and recognize one another (Clarke, 2013), and observers more generally use clothing and accessories to sort people into social categories (Hayfield, 2013; Carroll and Gilroy, 2002; Clarke, 2013). In Bangladesh, female students in particular are navigating this terrain under its own local pressures, balancing modesty expectations against contemporary fashion. This is not unique to Bangladesh. Research on young consumers in other socially restrictive societies finds similar patterns of hybrid styling, layering, and small acts of customization that let people express identity while staying inside the boundaries their community sets (Abid et al., 2026). Read against that work, Bangladeshi students' navigation of modesty and fashion looks less like an isolated cultural quirk and more like one local version of a broader pattern: identity expressed through negotiated, constrained agency rather than free personal choice.

Taken together, the literature makes a reasonably strong case that clothing affects confidence from both the inside and the outside, but almost never asks whether those two directions are separate in a collectivist setting, or whether they run together. Existing measures also tend to blur self-expression, social signaling, and performance beliefs into one another (Kwon, 1991; Peluchette and Karl, 2007), which makes the question harder to answer with the tools currently available. Mixed-methods work that could explain not just whether but how and why these effects occur remains rare in this context, and the growing pull of social media and fast fashion on youth identity in Bangladesh is barely studied at all. Building on this, the present study treats clothing-related confidence as a construct with two theorized pathways, set out below, whose degree of separateness is itself an open empirical question rather than a starting assumption (see Table 1).

**Table 1:** Conceptual dimensions of clothing-related confidence and their primary pathways.

Construct	Definition	Primary pathway
Self-expression and performance confidence	The internal sense of confidence individuals experience when clothing aligns with their identity, comfort, authenticity, or perceived readiness for academic and social tasks.	Internal (enclothed cognition, self-perception)
Social identity and external influence confidence	The confidence shaped by social evaluation, peer perception, cultural expectations, and media influence.	External (impression management, social feedback)

Conceptually, these two pathways are distinct. But if self-construal theory is right about how collectivist cultures construct the self, they may not stay as distinct empirically as their Western theoretical origins would suggest. The present study investigates exactly this, examining how internal and external pathways jointly, and perhaps jointly to a greater degree than existing theory anticipates, shape clothing-related confidence among Bangladeshi university students.

## Research methodology

### Research design

This study employed a sequential explanatory mixed-methods design (Plano Clark, 2017), integrating quantitative and qualitative approaches to examine the influence of clothing on self-confidence among university students in Bangladesh (Creswell and Clark, 2017; Karell and Niinimäki, 2020). Guided by a pragmatic research philosophy, the study combined a post-positivist quantitative phase to identify statistical relationships with a constructivist qualitative phase to interpret these findings through participants lived experiences.

The quantitative phase provided quantitative estimates for the recruited sample regarding the relationship between clothing and confidence, while the qualitative phase offered deeper contextual understanding of how and why clothing influences psychological experiences within a specific socio-cultural context (Guetterman et al., 2019).

### Sampling and data collection: quantitative phase

Data were collected using an online survey administered to university students across Bangladesh. A stratified convenience sampling approach was employed to ensure variation across gender, institution type (public and private), and year of study. The survey was distributed via Google Forms through email and social media platforms in July 2025. Participation was voluntary, and respondents were informed about the purpose of the study and the confidentiality of their responses prior to participation. The questionnaire required approximately 10–12 min to complete. A total of 312 valid responses were obtained. The sample consisted of 72.4% male and 27.6% female participants, with the majority enrolled in public universities (94.6%). Respondents represented all academic years, with the largest proportion of fourth-year students (44.2%).

### Measurement development and instrumentation

The questionnaire consisted of three sections. Section A collected demographic information (age, gender, institution type, and year of study). Sections B and C included 13 Likert-scale items measured on a 5-point scale (1 = strongly disagree to 5 = strongly agree), designed to assess clothing-related self-confidence.

The measurement items were developed using a theory-informed approach. Concepts from enclothed cognition (Adam and Galinsky, 2012), impression management (Peluchette and Karl, 2007), and cultural clothing practices in Bangladesh (Mahamud, 2016; Minhus and Huie, 2021) were used to guide the identification of key dimensions related to clothing and confidence. In addition, prior research on the functional aspects of clothing such as comfort, assurance, fashion, and individuality (Kwon and Parham, 1994) informed the conceptual framing of the items.

Based on these theoretical foundations, items were constructed to capture multiple aspects of clothing-related confidence, including self-expression, perceived performance, and social influence. The wording of the items was adapted to reflect the context of Bangladeshi university students. The item pool was developed in consultation with disciplinary experts and piloted with 10 university students prior to data collection.

### Validation of the measurement scale

The psychometric properties of the instrument were assessed using exploratory factor analysis (EFA) followed by confirmatory factor analysis (CFA). The EFA identified a two-factor structure explaining 49.15% of the total variance. Items were retained based on primary factor loadings (≥0.40), cross-loadings (<0.40 where possible), theoretical relevance, and overall conceptual interpretability of the factor structure. To validate this structure, CFA was conducted using a split-sample approach. The results indicated an acceptable model fit (CFI ≥ 0.95, RMSEA ≤ 0.06).

Internal consistency was evaluated using Cronbach's alpha. The overall scale demonstrated good reliability (α = 0.866), while the Self-Expression subscale showed strong internal consistency (α = 0.83). The Social Identity subscale exhibited lower reliability (α = 0.59), indicating that the items within this factor may not fully capture a single cohesive construct. This may reflect the multifaceted and context-dependent nature of social influences on clothing-related confidence. Accordingly, this factor is interpreted with caution and considered exploratory, suggesting the need for further refinement and expansion of items in future research.

### Additional survey items

A gender-specific item was included to assess the perceived influence of traditional/formal attire on confidence. Using conditional branching in the survey design, male respondents were presented with a version referring to panjabi-pajama or suit, while female respondents were presented with a version referring to saree. Responses were collected using a three-point categorical scale (Yes/No/Maybe). This item was analyzed separately and was not included in the factor analysis. An optional open-ended question was also included to capture additional qualitative insights regarding how clothing influences confidence and social perception.

### Sampling and data collection: quantitative phase

To complement the quantitative findings, qualitative data were collected through semi-structured interviews with a purposive sub-sample of 22 students (14 male, 8 female), selected to capture variation in gender and clothing preferences. In addition, responses from the optional open-ended survey question provided supplementary qualitative insights. A semi-structured interview guide was designed to explore participants lived experiences and personal perceptions of how clothing influences self-confidence. The guide was informed by the study's research objectives and relevant literature on enclothed cognition, impression management, and cultural clothing practices.

The interview protocol consisted of 10 open-ended questions organized into four thematic areas: general clothing preferences, confidence in academic and social settings, social and cultural influences, and reflections on mental wellbeing. The semi-structured format allowed flexibility for probing and follow-up questions, enabling participants to provide detailed narratives. Interviews were conducted in August 2025, lasted approximately 30–45 min, and were carried out in participants' preferred language (English or Bengali). All interviews were audio-recorded and transcribed verbatim.

## Data analysis

### Quantitative data

Quantitative data were analyzed using IBM SPSS Statistics (Version 28; IBM Corp., Armonk, NY, USA). Descriptive statistics (means, standard deviations, and frequencies) were calculated, followed by inferential analyses including independent-samples *t*-tests, chi-square tests, Pearson correlations, and hierarchical multiple regression to examine predictors of confidence (Field, 2024; Tabachnick and Fidell, 2019). Missing data was minimal across items (<1% per item) and were handled using full information maximum likelihood (FIML) for CFA models and listwise or pairwise deletion as appropriate for other analyses, consistent with each procedure's requirements.

Exploratory factor analysis (EFA) was conducted to identify underlying latent structures. Confirmatory factor analysis (CFA) was then performed using a split-sample approach (*N* = 312; *n* = 156 per group) in JASP's structural equation modeling module. Models were estimated using robust maximum likelihood (MLR). Model fit was evaluated using χ^2^ (df), CFI, TLI, RMSEA (90% confidence interval), and SRMR. Standardized factor loadings and modification indices were examined.

### Qualitative data

Qualitative data were analyzed using thematic analysis following the six-phase framework of Braun and Clarke (2006): (1) familiarization with the data, (2) generating initial codes, (3) identifying themes, (4) reviewing themes, (5) defining and naming themes, and (6) producing the final report. NVivo (Lumivero, Denver, CO, USA) software was used to support coding and theme organization. To enhance reliability, a subset of the data (25%) was independently coded, achieving strong inter-coder agreement (Cohen's κ > 0.80). Any discrepancies were resolved through discussion to ensure consistency and credibility.

## Results and analysis

### Quantitative analysis

Table 2 presents the demographic characteristics of the participants, including age, gender, university type, and year of study. The overall reliability coefficient was α = 0.866, indicating a high level of internal consistency (see Table 3) (α > 0.80 is considered good; George and Mallery, 2024).

**Table 2:** Demographic characteristics of participants (N = 312).

Age Group	*N*	%
18–20	11	3.5%
21–23	163	52.2%
24+	138	44.2%
Gender		
Male	226	72.4%
Female	86	27.6%
University Type		
Public	295	94.6%
Private	17	5.4%
Year of study		
1st year	32	10.3%
2nd year	62	19.9%
3rd year	76	24.4%
4th year	142	45.5%

**Table 3:** Reliability statistics for clothing-confidence scale.

Cronbach's alpha	Cronbach's alpha based on standardized items.	No of items	Interpretation
0.866	0.872	13	Good internal consistency

As shown in Table 2, a total of 312 university students participated in this study, where the majority of participants were between 21–23 years of age (52.2%), followed by those aged 24 and above (44.2%). Most respondents were male (72.4%) and enrolled in public universities (94.6%). Nearly half of the sample were in their fourth year of study (45.5%), with smaller proportions in third year (24.4%), second year (19.9%), and first year (10.3%).

Table 4 presents the descriptive statistics of the Likert-scale items measuring the relationship between clothing and self-confidence among university students.

**Table 4:** Descriptive statistics of likert-scale items (N = 312).

Item	*N*	Mean (*M*)	SD
I feel more confident when I wear clothes I like.	312	4.47	0.83
I dress differently for academic and social settings.	310	4.17	1.01
Wearing neat or formal clothes makes me feel more serious or motivated.	312	4.47	0.87
I choose clothes that reflect my personal identity and personality.	312	4.32	0.93
My clothing choices are influenced by social media or fashion trends.	312	2.95	1.33
My clothing affects how confident I feel in class or public settings.	312	3.74	1.16
I perform better academically when I am confident about my appearance.	312	3.46	1.27
I feel more socially accepted when I wear clothes that match the group.	310	3.61	1.19
I have been judged or complimented based on my attire.	312	3.69	1.16
My clothing choices can influence my mental wellbeing or self-image.	312	4.04	1.03
I feel less confident when I am forced to wear clothes I dislike.	312	4.04	1.15
Formal or professional clothes make me feel confident in interviews.	312	4.34	0.89
My clothing choice affects my performance in competitive settings (e.g., presentations, debates).	312	4.07	1.01

The scale was from 1 (Strongly Disagree) to 5 (Strongly Agree). Interpretation based on mean scores: 4.50–5.00 = Very Strong Agreement, 4.00–4.49 = Strong Agreement, 3.50–3.99 = Agreement, 3.00–3.49 = Neutral / Slight Agreement, 2.00–2.99 = Disagreement, 1.00–1.99 = Strong Disagreement. *N* values differ slightly due to individual item non-response.

While some descriptive patterns may appear intuitive, they provide an important baseline for understanding the more complex psychological, cultural, and social relationships examined in the subsequent analyses.

To assess the internal consistency of the 13 Likert-scale items measuring the relationship between clothing and self-confidence, Cronbach's alpha was calculated. The overall reliability coefficient was α = 0.866, indicating a high level of internal consistency (α > 0.80) is considered good (George and Mallery, 2024).

Corrected item–total correlations (CITC) ranged from 0.326 to 0.653, showing that each item was adequately correlated with the overall scale. Additionally, the “Cronbach's Alpha if Item Deleted” values ranged between 0.849 and 0.870, none of which were substantially higher than the overall alpha. This suggests that all items contributed meaningfully to the scale, and removing any item would not improve reliability. Therefore, the scale demonstrates strong reliability and internal consistency. To examine whether gender influences perceived confidence from traditional/formal attire, responses to Q18 were cross tabulated by gender (Table 5).

**Table 5:** Crosstabulation of gender and clothing-related confidence (Q18).

Response	Male (*n* = 218)	%	Female (*n* = 86)	%	Total (*N* = 304)	%
No	12	5.5	10	11.6	22	7.2
Maybe	56	25.7	42	48.8	98	32.2
Yes	150	68.8	34	39.5	184	60.5

The gender-specific items were analyzed separately to provide contextual insights into cultural clothing preferences. These items were not included in the measurement model but offer additional support for the role of cultural attire in shaping confidence. A chi-square test of independence indicated this association was statistically significant, χ^2^(2, *N* = 304) = 22.18, *p* < 0.001, with a moderate effect size (Cramer's *V* = 0.27; see Table 6).

**Table 6:** Association between gender and clothing-related confidence (Q18).

Test (statistic)	*χ^2^*	df	N	*p* (2-tailed)	Cramer's *V* (effect size)
Pearson Chi-Square	22.18	2	304	<0.001	0.27

0 cells (0.0%) had expected counts <5; minimum expected count = 6.22. *N* = 304 (valid cases).

As shown in Table 5, a majority of male students (68.8%) reported “Yes,” indicating that wearing Panjabi–Pajama or suits increased their confidence, compared to 39.5% of females who felt more confident in Saree. Interestingly, nearly half of the females (48.8%) responded “Maybe,” while only 25.7% of males gave the same response. A small proportion of males (5.5%) and females (11.6%) reported “No.” Eight male respondents did not answer the attire-confidence item and were excluded listwise, yielding a valid analytic sample of *N* = 304 (218 male, 86 female) for this analysis.

A chi-square test of independence indicated this association was statistically significant, χ^2^ (2, *N* = 304) = 22.18, *p* < 0.001, with a moderate effect size (Cramer's *V* = 0.27). Note that the two groups were not asked an identical question: male respondents rated confidence wearing formal Western attire (e.g., suit) or Panjabi-pajama, while female respondents rated confidence wearing saree, reflecting gender-typical formal dress in this cultural context. The chi-square result therefore reflects differing responses to gender-specific, non-equivalent prompts, and should not be interpreted as a pure, unconfounded gender effect on attire confidence.

An exploratory factor analysis (EFA) was conducted using Principal Axis Factoring with Varimax rotation to identify the latent dimensions of clothing's impact on students' self-confidence. The Kaiser-Meyer-Olkin (KMO) measure was 0.903, and Bartlett's Test of Sphericity was significant (χ^2^ (78) = 1,298.59, *p*<*0.001*), indicating excellent suitability for factor analysis (Table 7). Two factors with eigenvalues greater than 1 were extracted, explaining 49.15% of the total variance (Table 8). The oblique Promax-rotated pattern matrix revealed two clear dimensions: Factor 1 (Self-Expression and Performance Confidence), on which items related to personal identity, formal attire, and interview readiness loaded most strongly; and Factor 2 (Social Identity and External Influence Confidence), on which items related to social acceptance and being judged on attire loaded most strongly (Table 9).

**Table 7:** Kaiser–Meyer–Olkin measure of sampling adequacy and Bartlett's test of sphericity.

Kaiser-Meyer-Olkin measure of sampling adequacy		0.903
Bartlett's test of sphericity	χ^2^	1,298.587
	df	78
	Sig.	0.000

KMO, Kaiser–Meyer–Olkin measure of sampling adequacy. A KMO value of 0.903 indicates excellent sampling adequacy. Bartlett's test of sphericity was statistically significant, χ^2^ (78) = 1,298.59, *p* < 0.001, indicating that the correlation matrix was suitable for factor analysis.

**Table 8:** Total variance explained (principal axis factoring).

Factor	Eigenvalue	% of variance	Cumulative %
1	5.23	40.25%	40.25%
2	1.16	8.90%	49.15%

Extraction method = Principal Axis Factoring. Factors retained according to eigenvalues > 1.

**Table 9:** Pattern matrix coefficients, communalities, and item retention (Promax rotation).

Item	Factor 1	Factor 2	Communality	Retained
I feel more confident when I wear clothes I like.	0.842	−0.302	0.8	Yes
I dress differently for academic and social settings.	0.702	−0.105	0.504	Yes
Wearing neat or formal clothes motivates me.	0.771	−0.029	0.595	Yes
I choose clothes that reflect my personality.	0.805	−0.163	0.675	Yes
My clothing choices are influenced by social media.	0.06	0.639	0.412	Yes
Clothing affects confidence in class/public.	0.591	0.215	0.396	No
I perform better academically with confidence.	0.445	0.31	0.294	No
I feel socially accepted when matching group dress.	−0.061	0.822	0.679	Yes
I have been judged/complimented on attire.	−0.198	0.762	0.62	Yes
Clothing influences my mental wellbeing.	0.601	0.197	0.4	Yes
Wearing disliked clothes lowers confidence.	0.49	0.15	0.263	No
Formal/professional clothes boost interview confidence	0.585	0.154	0.366	Yes
Clothing affects performance in competitions.	0.538	0.241	0.348	Yes

Extraction = Principal Axis Factoring. Rotation = Promax (oblique), used because the two factors are correlated (ϕ = 0.512) rather than orthogonal, as assumed by the original Varimax rotation. Items marked “No” were dropped from the final scale: the academic-performance-belief and dislike-of-clothing items showed low communality (<0.30) and/or ambiguous cross-loading; the class-confidence item was excluded on conceptual-redundancy grounds (overlap with the formal-attire-motivation item) for scale parsimony.

To provide a more rigorous test of factor retention beyond the conventional eigenvalue-greater-than-one rule, parallel analysis (1,000 random-data simulations) was conducted (see Table 10). This procedure suggested retention of a single factor, whereas the eigenvalue criterion and theoretical model supported two factors. Given this divergence, discriminant validity between the two factors was further examined using the heterotrait-monotrait ratio (HTMT). The HTMT value (0.696) fell well below the conservative 0.85 threshold, indicating that the two factors are empirically distinguishable despite their shared variance. Considered together with the acceptable-to-excellent CFA fit indices reported in Table 11, this pattern supports retaining the two-factor structure, while acknowledging that the constructs are closely related and may benefit from further refinement in future research.

**Table 10:** Comparison of eigenvalues: actual data vs. parallel analysis (random data).

Factor	Eigenvalue (actual data)	Mean eigenvalue (random data)	95th percentile eigenvalue (random data)	Retain?
1	5.23	1.36	1.44	Yes
2	1.16	1.27	1.33	No

Parallel analysis based on 1,000 random-data simulations matching the actual sample (*N* = 308, 13 variables). A factor is retained only if its actual eigenvalue exceeds the 95th percentile eigenvalue from random data. The conventional eigenvalue-greater-than-one criterion (Table 8) supported two factors; parallel analysis supported one. See narrative below for the discriminant-validity (HTMT) analysis that informed the final retention decision.

**Table 11:** Confirmatory factor analysis fit indices for the two-factor clothing self-confidence model.

Model	*n*	*χ^2^*	df	*p*	CFI	TLI	RMSEA	90% CI (RMSEA)	SRMR
Sample A (validation)	156	53.7	34	0.017	0.94	0.92	0.061	(0.030, 0.088)	0.052
Sample B (validation)	156	32.28	34	0.552	1	1.01	0	(0.00, 0.051)	0.043
Full sample (robustness)	312	58.75	34	0.005	0.96	0.94	0.048	(0.029, 0.067)	0.039

CFI, comparative fit index; TLI, Tucker-Lewis index; RMSEA, root mean square error of approximation; SRMR, standardized root mean square residual. All models were estimated using maximum likelihood robust estimation.

These findings suggest that students' clothing choices influence self-confidence across two main domains: performance-oriented contexts and social identity-related contexts. Because all variables were collected via self-report from one questionnaire at one time point, Harman's single-factor test was conducted to check for common method bias. All 13 items were constrained to a single, unrotated factor, which explained 40.25% of total variance, below the 50% threshold associated with a substantial bias concern (Podsakoff et al., 2003). This suggests common method bias is unlikely to substantially affect the results, though this test alone does not rule it out entirely.

To ensure reliability of the derived factors, Cronbach's alpha was calculated. Table 12 presents the descriptive statistics and reliability coefficients for each factor.

**Table 12:** Reliability and descriptive statistics of composite factors.

Factor	No. of items	*M*	SD	α
Self-expression and performance confidence	7	4.3	0.7	0.8
Social identity and external influence	3	3.4	0.9	0.6

As shown in Table 12, the Self-Expression and Performance Confidence factor demonstrated good reliability (α = 0.83). The high mean score (*M* = 4.27, *SD* = 0.66) indicates strong participant agreement that clothing enhances their personal confidence, motivation, and task performance.

In contrast, the Social Identity and External Influence factor yielded a lower reliability coefficient (α = 0.59), which falls below the conventional threshold of 0.70. This is not uncommon for short scales and may be attributed to the limited number of items and the conceptual breadth of the social influence construct (Pallant, 2020). Despite this, the moderate mean score (*M* = 3.42, *SD* = 0.91) suggests that participants endorsed, to a varying degree, the role of social approval, judgment, and media trends in shaping their clothing-related confidence.

Confirmatory factor analysis was conducted on the two-factor model using an internal cross-validation approach. The sample was randomly divided into two halves (Sample A and Sample B, *n* = 156 each), and the two-factor model was tested on both validation samples and on the full sample. As shown in Table 11, Sample A demonstrated acceptable fit (CFI = 0.94; TLI = 0.92; RMSEA = 0.061; SRMR = 0.052), Sample B showed excellent fit (CFI = 1.00; RMSEA = 0.000; SRMR = 0.043), and the full-sample CFA confirmed model stability (CFI = 0.955; RMSEA = 0.048; SRMR = 0.039).

A one-factor alternative was also tested on the same analytic sample (*N* = 308) used for the exploratory factor analysis and fit significantly worse than the two-factor model, Δχ^2^ (1) = 33.89, *p* < 0.001 (one-factor: CFI = 0.914, RMSEA = 0.081; two-factor: CFI = 0.954, RMSEA = 0.061), supporting the two-factor structure over a unidimensional alternative.

A second-order factor model, with a single higher-order construct explaining the two first-order factors, was also tested. With only two first-order factors, this model is mathematically equivalent to the freely correlated two-factor model already reported (identical χ^2^ = 72.41); a second-order model becomes empirically distinguishable only with three or more first-order factors. The two-factor correlated model was therefore retained for parsimony, consistent with the discriminant validity evidence reported above (HTMT = 0.696). Standardized factor loadings for the confirmatory subsample (Sample B) are presented in Table 13.

**Table 13:** Standardized factor loadings from CFA (Sample B, n = 156).

Item (variable)	Items	Factor	β	SE	*p*
Q5_confLike	I feel more confident when I wear clothes I like	SelfExpression	0.44	0.14	0.002
Q6_diffDress	I dress differently for academic/social settings	SelfExpression	0.5	0.09	<0.001
Q7_formalMot	Wearing neat/formal clothes makes me motivated	SelfExpression	0.75	0.05	<0.001
Q8_idReflect	I choose clothes that reflect my personality	SelfExpression	0.53	0.11	<0.001
Q14_mental	Clothes influence my mental wellbeing	SelfExpression	0.69	0.06	<0.001
Q16_interview	Formal clothes make me confident in interviews	SelfExpression	0.75	0.06	<0.001
Q17_CloAffectCompetition	Clothing affects confidence in competitions	SelfExpression	0.66	0.07	<0.001
Q9_socialMedia	Clothing choices influenced by social media	SocialIdentity	0.5	0.09	<0.001
Q12_socialAccept	I feel more accepted when clothes match the group	SocialIdentity	0.56	0.1	<0.001
Q13_judged	I have been judged/complimented based on my attire	SocialIdentity	0.52	0.11	<0.001

Std. loading, standardized factor loading (β). SE, standard error. All loadings are significant at *p* ≤ 0.002 (Q5) or *p* < 0.001 (others). Values rounded to two decimals.

The standardized factor loadings for the confirmatory sample (Sample B) are presented in Table 13. All loadings were statistically significant at *p* ≤ *0.002* (Q5) or *p*<*0.001* (others) and demonstrated adequate to strong relationships with their respective factors. The Self-Expression factor showed particularly robust loadings for clothing motivation (Q7_formalMot, β = 0.75) and interview confidence (Q16_interview, β = 0.75). The Social Identity factor exhibited moderate loadings, with social acceptance (Q12_socialAccept, β = 0.56) and social judgment (Q13_judged, β = 0.52) demonstrating the strongest associations. The two-factor model is illustrated in Figure 1 (path diagram from Sample B).

**Figure 1:**
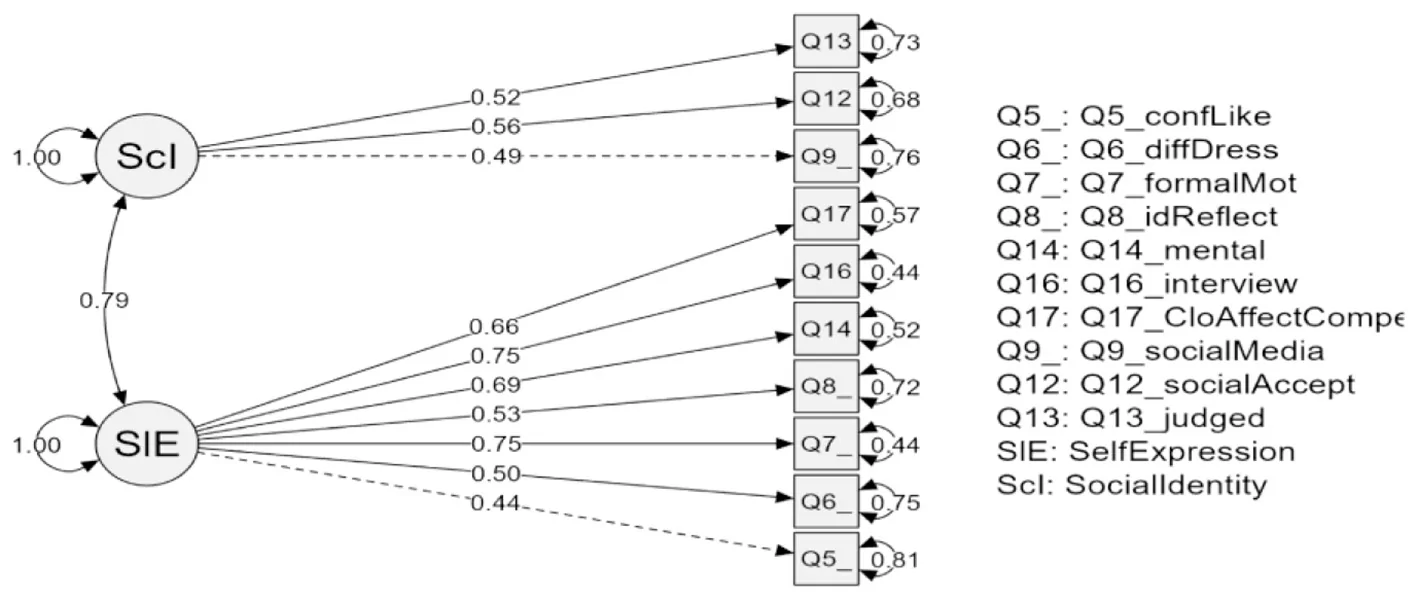
Path diagram of the two-factor clothing self-confidence model with standardized loadings. In this figure the path diagram of the two-factor Clothing Self-Confidence model (Sample B: confirmatory subsample). Standardized factor loadings (β) are shown on single-headed arrows; numbers beside item rectangles are error variances. The double-headed arrow shows the latent-factor correlation (*r* = 0.79). Dashed lines indicate the two lowest-magnitude standardized loadings (Q5, Q9). Numeric loadings correspond to those reported in Table 13.

Several psychometric observations warrant consideration. Some items demonstrated moderate conceptual overlap across the two dimensions, suggesting that certain aspects of clothing-related confidence may simultaneously reflect internal self-expression and socially mediated confidence processes. In addition, the high latent factor correlation (*r* = 0.79) indicates that the two factors are strongly interconnected and may represent related dimensions of a broader clothing-related confidence construct. Item retention decisions were based on primary factor loadings, theoretical relevance, and overall conceptual interpretability of the factor structure. Nevertheless, the relatively lower reliability of the Social Identity and External Influence factor suggests that this dimension should be interpreted cautiously and further refined in future research.

Composite reliability (CR) and average variance extracted (AVE), computed from the full-sample standardized CFA loadings, are reported in Table 14. SelfExpression showed acceptable composite reliability (CR = 0.83) but modest convergent validity (AVE = 0.42), indicating the factor explains about 42% of the variance in its indicators. SocialIdentity displayed lower composite reliability (CR = 0.60) and AVE = 0.34, suggesting more limited convergent validity for that subscale. McDonald's omega, considered a more appropriate reliability estimate than Cronbach's alpha for a multidimensional scale, was consistent with the CR estimates (ω = 0.83 for Self-Expression; ω = 0.61 for Social Identity). The baseline (configural) model showed acceptable fit for male respondents (*n* = 223; CFI = 0.92, RMSEA = 0.085) but did not meet conventional fit thresholds for female respondents (*n* = 85; CFI = 0.86, RMSEA = 0.10; see Table 15). Given that AVE fell below the conventional 0.50 threshold for both factors, and Social Identity's reliability remains marginal, the two-factor solution is best regarded as provisional pending refinement of the Social Identity subscale in future research; substantive claims relying on this subscale alone should be interpreted with appropriate caution.

**Table 14:** Composite reliability and convergent validity for the two-factor model (N = 312).

Factor	No. of items	Composite reliability (CR)	McDonald's omega (ω)	Average variance extracted (AVE)
SelfExpression	7	0.83	0.83	0.42
SocialIdentity	3	0.6	0.61	0.34

CR, composite reliability; ω, McDonald's omega, computed from standardized CFA loadings, recommended over Cronbach's alpha for multidimensional scales. CR/ω ≥ 0.70 indicates acceptable construct reliability; AVE ≥ 0.50 indicates acceptable convergent validity.

**Table 15:** Configural measurement invariance fit by gender.

Group	*n*	*χ^2^*	df	CFI	TLI	RMSEA
Male	223	88.16	34	0.916	0.889	0.085
Female	85	62.68	34	0.863	0.819	0.1

Configural (baseline, unconstrained) model fit estimated separately by gender. Because configural fit did not meet conventional thresholds in both groups, metric and scalar invariance were not tested.

Prior to testing gender differences in composite scores, we evaluated measurement invariance across gender. The baseline (configural) model showed acceptable fit for male respondents (*n* = 223; CFI = 0.92, RMSEA = 0.085) but did not meet conventional fit thresholds for female respondents (*n* = 85; CFI = 0.86, RMSEA = 0.10), likely reflecting the smaller female subsample relative to model complexity. Because configural fit was not adequate in both groups, formal tests of metric and scalar invariance were not pursued, as constraining parameters on a poorly fitting baseline would not yield interpretable results. Gender comparisons reported below should therefore be interpreted as differences in observed composite scores rather than latent factor means, consistent with recommendations for cases where invariance cannot be established.

An independent-samples *t*-test was conducted to evaluate the impact of gender on the two dimensions of clothing-related confidence. The results, summarized in Table 16, revealed a statistically significant gender difference for the Self-Expression and Performance Confidence factor.

**Table 16:** Independent samples T-test results for gender differences.

Factor	Gender	*N*	*M*	SD	*t*	df	*p*	Cohen's *d*
Self-expression	Male	226	4.19	0.69	−3.48	310	0.001	0.45
	Female	86	4.48	0.52				
Social influence	Male	226	3.39	0.93	−0.75	310	0.454	0.1
	Female	86	3.48	0.87				

*p*-values are two-tailed. Cohen's *d* is reported in absolute value.

Contrary to what might be assumed, female students (*M* = 4.48, SD = 0.52) reported significantly higher levels of confidence derived from self-expression and performance in their clothing choices compared to male students (*M* = 4.19, SD = 0.69), t (310) = −3.48, *p* = 0.001. The magnitude of the difference in the means was medium (Cohen's *d* = 0.45).

In contrast, there was no significant difference between genders on the Social Identity and External Influence factors (*p* = 0.454). Both male (*M* = 3.39, SD = 0.93) and female (*M* = 3.48, SD = 0.87) students reported moderate and statistically similar levels of influence from social compliance and external judgment. Descriptive statistics were computed to examine mean levels of self-expression and social influence confidence across years of study and gender (Table 17).

**Table 17:** Means and standard deviations for self-expression and social influence confidence by year of study and gender.

Year of study	Gender	^*^ *n* ^*^	Self-expression M, (SD)	Social influence M, (SD)
1st year	Male	22	4, (1.02)	3.24, (1.1)
	Female	10	4.59, (0.47)	3.3, (0.91)
2nd year	Male	43	4.25, (0.61)	3.17, (1.05)
	Female	19	4.39, (0.72)	3.4, (1.1)
3rd year	Male	60	4.23, (0.7)	3.65, (0.93)
	Female	16	4.44, (0.52)	3.6, (0.86)
4th year	Male	101	4.18, (0.63)	3.37, (0.81)
	Female	41	4.51, (0.43)	3.51, (0.75)
Total	Male	226	4.19, (0.69)	3.39, (0.93)
	Female	86	4.48, (0.52)	3.48, (0.87)
Grand total		312	4.27, (0.66)	3.42, (0.91)

Self-expression and social influence were measured on a 5-point Likert scale (1 = Strongly Disagree, 5 = Strongly Agree).

As shown in Table 17, mean self-expression and social influence scores are presented by year of study and gender. To test whether these patterns were statistically significant, a two-way ANOVA was conducted with gender and year of study as independent variables (see Table 18).

**Table 18:** Two-way anova results for self-expression and social influence.

Dependent variable	Source	df	*F*	*p*	Partial *η^2^*
Self-expression	Year of study	3, 304	0.81	0.487	0.008
	Gender	1, 304	5.54	0.019	0.018
	Yr × Gen	3, 304	0.78	0.506	0.008
Social influence	Year of study	3, 304	2.68	0.047	0.026
	Gender	1, 304	0.03	0.868	<0.001
	Yr × Gen	3, 304	0.22	0.88	0.002

### Self-expression

A significant main effect of gender emerged, F (1, 304) = 5.54, *p* = 0.019, partial η^2^ = 0.018, with female students reporting higher self-expression confidence than male students (*M* = 4.48 vs. *M* = 4.19). Neither the main effect of year of study, F (3, 304) = 0.81, *p* = 0.487, nor the Gender × Year interaction, F (3, 304) = 0.78, *p* =0.506, was significant. Group means across years were essentially flat (Year 1 M = 4.18 to Year 4 M = 4.28), showing no evidence of a rising trend across academic years.

### Social influence

Year of study showed a small but significant main effect, F (3, 304) = 2.68, *p* = 0.047, partial η^2^ = 0.026. However, Tukey *post-hoc* comparisons found no individual pairwise difference between years reached significance after correction (all p_adj > 0.05), indicating this effect is weak and should be interpreted with caution rather than attributed to a specific academic-year transition. Gender was not significant, F (1, 304) = 0.03, *p* = 0.868, nor was the Gender × Year interaction, F (3, 304) = 0.22, *p* = 0.880.

Gender differences were evident in self-expression confidence, with female students consistently reporting higher scores, while no significant differences emerged for social influence across gender or year of study. To further examine the relationships among the key constructs, a Pearson correlation analysis was conducted between the two composite factors (Self-Expression Confidence and Social Identity Confidence) and the single-item measures of appearance-related academic-performance belief and social media influence.

As shown in Table 19, Self-Expression Confidence was positively correlated with the appearance-related academic-performance belief item (*r* = 0.53, *p* < 0.01) and with Social Media Influence (*r* = 0.40, *p* < 0.01), indicating that students who feel confident in their clothing choices also report stronger appearance-performance beliefs and are more attentive to social-media fashion trends.

**Table 19:** Pearson correlations between key study variables (N = 312).

Variable	1	2	3	4
Self-expression confidence	—			
Social identity confidence ^a^	0.43^**^	—		
Appearance-related academic-performance belief (Q11)	0.53^**^	0.34^**^	—	
Social media influence (Q9)	0.40^**^	0.39^**^	0.31^**^	—

Values are Pearson correlation coefficients. *p* < 0.01 (2-tailed). ^a^ Social Identity Confidence here is computed excluding Q9 (mean of Q12 and Q13 only) to avoid part-whole overlap with the Social Media Influence item, consistent with sensitivity-analysis recommendations. ^**^indicates *p* < 0.01.

Because Q9 (Social Media Influence) is one of the three indicators of the Social Identity Confidence factor, the composite used in this table was recalculated excluding Q9 to avoid a part-whole correlation. Using this corrected composite, Social Identity Confidence showed a moderate correlation with Social Media Influence (*r* = 0.39, *p* < 0.01), comparable in size to its correlation with Self-Expression (*r* = 0.43) and the performance-belief item (*r* = 0.34) indicating no evidence of disproportionate overlap between the constructs.

Prior to interpreting the regression results, it is important to clarify the conceptual distinction between the predictor variables and the dependent construct. The item measuring appearance-related academic-performance belief (Q11: “I believe I perform better academically when I am confident about my appearance”) reflects a belief regarding the perceived impact of appearance on academic performance, whereas the dependent variable, Self-Expression Confidence, represents a broader psychological confidence state associated with clothing. Although these constructs are conceptually related, they are not identical. The moderate cross-loadings observed in the EFA suggest partial conceptual overlap, which is theoretically expected given the interconnected nature of clothing-related psychological experiences. Including Q11 in the regression model therefore allowed the analysis to examine whether performance-related beliefs contributed additional explanatory variance beyond general self-expression confidence. Nevertheless, potential shared method variance and conceptual overlap should be acknowledged when interpreting the regression results. Future research may benefit from incorporating more distinct psychological constructs or objective performance indicators to further strengthen construct separation. A hierarchical multiple regression was conducted to assess the contribution of psychological factors to self-expression confidence beyond demographic variables (see Table 20).

**Table 20:** Hierarchical regression predicting self-expression from demographics and psychological predictors.

Model	Predictor	B	SE B	β	t	p	95% CI for B
Model 1: demographics	Gender	0.287	0.082	0.194	3.48	0.001	(0.125, 0.449)
	Year of study	0.018	0.036	0.028	0.51	0.614	(−0.052, 0.088)
Model 2: psychological predictors	Gender	0.167	0.067	0.113	2.5	0.013	(0.035, 0.298)
	Year of study	−0.004	0.029	−0.007	−0.15	0.882	(−0.060, 0.052)
	Social influence	0.249	0.035	0.344	7.11	<0.001	(0.180, 0.318)
	Appearance-related academic-performance belief (Q11)	0.197	0.026	0.379	7.74	<0.001	(0.147, 0.247)

*N* = 312; CI, confidence interval.

For Step 1: *R*^2^ = 0.04, Adjusted *R*^2^ = 0.03, F (2, 309) = 6.16, *p* = 0.002.

For Step 2: *R*^2^ = 0.39, Adjusted *R*^2^ = 0.39, ΔR^2^ = 0.36, F (4, 307) = 49.96, *p* < 0.001.

As shown in Table 20, Demographic variables (gender and year of study) were entered in Model 1, explaining a significant but small proportion of the variance, *R*^2^ = 0.04, F (2, 309) = 6.16, *p* = 0.002. Gender was a significant positive predictor in this step (β = 0.19, *p* = 0.001), indicating that female students reported higher self-expression confidence.

The psychological constructs on social influence confidence and appearance-related academic-performance belief were added in Model 2. The addition of these constructs explained a substantial additional 35.6% of the variance, ΔR^2^ = 0.36, *p* < 0.001. The full model accounted for 39.4% of the total variance in self-expression confidence (Adjusted *R*^2^ = 0.39).

In the final model, appearance-related academic-performance belief was the strongest unique predictor (β = 0.38, *p* < 0.001), closely followed by social influence confidence (β = 0.34, *p* < 0.001). Gender remained a significant unique predictor (β = 0.11, *p* = 0.013), though its effect size was reduced, suggesting that gender's association with self-expression confidence is partially attenuated once psychological variables are included; this descriptive attenuation does not establish mediation. Year of study was not a significant predictor at any step.

### Qualitative findings

Thematic analysis of the 22 in-depth interviews and 183 open-ended survey responses revealed five interconnected themes that explain how Bangladeshi university students perceive the relationship between clothing and self-confidence. These themes provide depth to the statistical patterns observed in the quantitative analysis, highlighting the psychological, cultural, and social mechanisms through which clothing affects confidence. A summary is presented in Table 21.

**Table 21:** Emergent themes from thematic analysis of interviews and open-ended responses.

Theme	Description	Illustrative prevalence
The Primacy of Comfort and Authenticity	Confidence is rooted in wearing clothes that are physically comfortable, well-fitting, and true to one's personal style.	Ubiquitous across both genders
Context-Specific “Armor”	Formal or culturally significant attire (e.g., Panjabi, suit, saree) is used strategically to boost confidence in high-stakes settings such as presentations, interviews, or religious events.	Very high
The Weight of the Social Gaze	Students recognize that clothing shapes others' judgments; neatness and appropriateness strongly influence first impressions.	Very high
Self-Expression and Identity Negotiation	Clothing is a medium of self-expression and cultural identity, particularly salient for female students negotiating societal expectations.	High
Inner Self-Reliance (Counterpoint)	A minority emphasized that true confidence is internal, with clothing playing only a superficial role.	Low (but significant)

### Theme 1: the primacy of comfort and authenticity

The most consistent theme was the foundational role of comfort and authenticity. Participants explained that confidence stemmed not from fashionability but from feeling physically at ease and aligned with their personal style. Ill-fitting or uncomfortable clothing, by contrast, was linked to distraction and diminished confidence.

- “*When I feel so good to wear comfortable clothing, my confidence increases.”* (Male, open-ended response)
- “*Uncomfortable dress up makes my mind inattentive about my work… my mind [is] focused on my comfort.”* (Female, interview)

This finding underscores that comfort is a psychological prerequisite, forming the baseline upon which other clothing-related confidence effects operate.

### Theme 2: context-specific “armor”

Students often described clothing as a form of strategic “armor” for important occasions. Formal or traditional attire was reported to enhance feelings of preparedness, professionalism, and respectability in academic or social contexts.

- “*Wearing a suit or Panjabi for a job interview makes me feel more organized and responsible.”* (Male, open-ended response)
- “*When I wear a saree in a cultural program, it increases my confidence because I feel elegant and prepared.”* (Female, interview)

This aligns with the concept of **enclothed cognition**, as participants internalized the symbolic meanings of attire to boost psychological readiness.

### Theme 3: the weight of the social gaze

Participants expressed a strong awareness of how clothing influences others' perceptions. Neat, clean, and appropriate clothing was consistently linked to projecting competence and professionalism, thereby reinforcing self-confidence through positive feedback and impression management.

- “*First impression always comes from outfit.”* (Male, interview)
- “*People see me by my clothes, so if I wear clean and proper dress, they get a good impression.”* (Female, open-ended response)

Here, confidence was not only about internal feelings but also about anticipating external judgments and aligning dress to meet social expectations.

### Theme 4: self-expression and identity negotiation

Clothing also emerged as a medium of self-expression and identity. Students reported that attire reflecting personal taste or cultural heritage strengthened their sense of authenticity and boosted self-confidence. This theme was particularly pronounced among female students, who often described negotiating personal preferences with cultural norms of modesty.

- “*Our taste is expressed through clothing and attire... It carries the hallmark of elegance and sophistication.”* (Female, open-ended response)
- “*Wearing a burqa gives me confidence because it aligns with who I am and how my society sees me.”* (Female, interview)

This finding connects directly to the quantitative evidence that self-expression is a significant factor in overall confidence.

### Theme 5: inner self-reliance (counterpoint)

A minority of participants challenged the dominant narrative, arguing that true confidence originates from within rather than from clothing. They viewed attire as a surface-level factor, affecting only external impressions.

- “*Clothing doesn't impact my confidence… Confidence is about your mindset, mental health, and self-reliance.”* (Male, open-ended response)

Including this counter-narrative enriches the analysis by demonstrating variation in perspectives, highlighting that while clothing strongly influences confidence for most, it is not universally perceived as essential.

### Integration of quantitative and qualitative findings

Table 22 summarizes how quantitative patterns map onto emergent qualitative themes and their primary empirically derived factor relationships. Notably, Themes 1 (Comfort and Authenticity) and 4 (Identity Negotiation) operate across both latent dimensions, consistent with the within-factor performance association (*r* = 0.53) and the high between-factor correlation (*r* = 0.79).

**Table 22:** Integration of qualitative themes with empirically derived clothing confidence factors.

Quantitative finding	Qualitative theme	Primary factor (CFA)	Interpretation
Females (*M* = 4.48) reported higher self-expression confidence than males (*M* = 4.19), t (310) = −3.48, *p = 0.001*.	Theme 4: self-expression and identity negotiation	Self-expression and performance confidence	Female participants described more complex identity work balancing personal style with societal expectations, consistent with heightened clothing awareness.
High agreement: “Wearing neat/formal clothes makes me feel more serious or motivated” (*M* = 4.47).	Theme 2: context-specific “armor”	Self-expression and performance confidence	Students reported using formal attire strategically to enhance psychological preparedness and task focus, aligning with observed performance-confidence loadings (β ≈ 0.75).
Strong correlations: self-expression ↔ academic-performance belief (*r* = 0.53, *p <0.01*) and latent factor correlation (*r* = 0.79).	Theme 1: comfort and authenticity; Theme 4: identity negotiation	Both	Cross-cutting influences of comfort and identity negotiation explain both the internal performance link (self-expression → performance belief) and the strong interrelationship between confidence dimensions (self-expression ↔ social identity).
Social Identity Confidence correlated with social media influence (*r* = 0.77, *p <0.01*).	Theme 3: social gaze	Social identity and external influence	External judgments, peer evaluation, and media cues corresponds to strong social-identity effects, consistent with impression-management narratives.
Hierarchical regression: psychological constructs predicted self-expression (ΔR^2^ = 0.356, *p <0.001*).	Combined influence: Themes 2–4 (armor, social gaze, identity negotiation)	Both (primarily Self-Expression)	The block of themes jointly predicts self-expression beyond demographics; strategic dressing, social evaluation, and identity negotiation together account for substantial variance.
Minority perspective challenging clothing's importance.	Theme 5: inner self-reliance (counter-narrative)	Both (qualifying)	A smaller subgroup emphasized internal sources of confidence, qualifying the dominant pattern and indicating boundary conditions for clothing's impact.

Themes were derived from Table 21; the Primary Factor column maps each theme to the two empirically derived factors (Self-Expression and Performance Confidence; Social Identity and External Influence). Statistical values (*t, r*, ΔR^2^, *p*) are from analyses reported in Section Quantitative analysis. See Table 13 for standardized factor loadings (Sample B) and Table 11 for CFA fit indices. Exemplar quotations supporting each theme are provided in Section Qualitative findings.

To visually represent these integrated relationships, Figure 2 presents a conceptual model mapping the qualitative themes onto the empirically derived factor structure.

**Figure 2:**
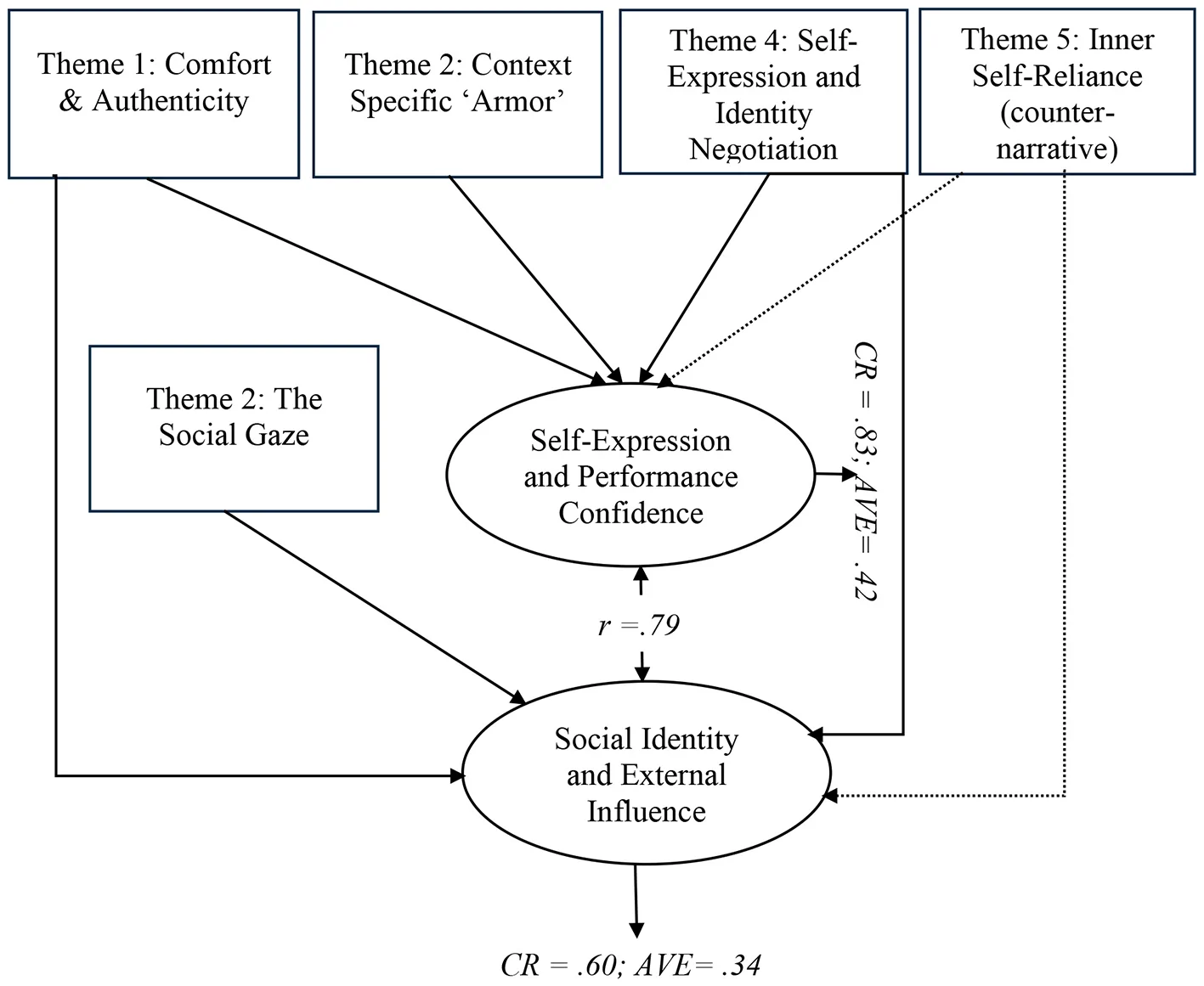
Integrated conceptual model of clothing-related self-confidence among Bangladeshi university students. The model integrates five qualitative themes with the CFA-validated two-factor measurement model. Solid arrows indicate primary thematic influences on each latent factor; dashed arrows indicate secondary or conditional influences (counter-narrative). The double-headed arrow shows the latent factor correlation (*r* = 0.79). CR, composite reliability; AVE, average variance extracted. See Table 11 for standardized factor loadings and Table 12 for fit indices.

Figure 2 represents an integrated model that maps the five emergent qualitative themes onto the CFA-validated two-factor structure of clothing-related self-confidence. The model illustrates that Self-Expression and Performance Confidence is primarily influenced by Theme 2 (Context-Specific “Armor”) and Theme 1 (Comfort and Authenticity), reflecting internal psychological processes. Social Identity and External Influence Confidence is principally driven by Theme 3 (Social Gaze) and Theme 4 (Self-Expression and Identity Negotiation), capturing external social dynamics. The high correlation between factors (*r* = 0.79) is explained by cross-cutting influences, particularly Theme 1 and Theme 4, which operate across both dimensions. Theme 5 (Inner Self-Reliance) serves as a qualifying counter-narrative, challenging the dominant pattern through dashed connections to both factors.

## Discussion

This mixed-methods study examined how clothing relates to self-confidence among Bangladeshi university students, combining survey data (*N* = 312), 22 semi-structured interviews, and 183 open-ended responses. Two interconnected quantitative dimensions emerged, Self-Expression and Performance Confidence and Social Identity and External Influence Confidence, and five qualitative themes helped explain how students actually experience these dimensions day to day: comfort and authenticity, clothing as context-specific “armor,” the social gaze, identity negotiation, and inner self-reliance. Rather than simply listing these results again, this section asks why they likely took this shape, how they sit against research conducted mostly in Western, individualist settings, and what that comparison reveals theoretically.

The item that received the strongest endorsement in the sample (*M* ≈ 4.47) was feeling more confident in preferred clothing, and formal or neat attire being linked to feeling more serious or motivated ran a close second. Read alongside the qualitative interviews, this looks less like a simple preference and more like clothing functioning as a cue student used to signal readiness to themselves before an academic or social task. This is consistent with enclothed cognition's central claim (Adam and Galinsky, 2012), though it is worth being precise about what the data can and cannot support here. Students reported feeling more prepared and confident; the study did not measure academic performance itself, so any language implying that clothing improved outcomes, rather than the felt sense of readiness for them, would overstate what was tested. The same caution applies to the item referencing mental wellbeing: what was captured is a belief that clothing affects wellbeing, not an independent measure of wellbeing itself.

Gender differences followed a similar pattern to what the regression and ANOVA results already showed. Female students reported significantly higher self-expression confidence than male students [t (310) = −3.48, *p* = 0.001], and this was echoed in the corrected two-way ANOVA, where gender was the only significant predictor of Self-Expression confidence [F (1, 304) = 5.54, *p* = 0.019] and year of study was not. The qualitative interviews offer one plausible account of why: several female participants described actively balancing personal style against modesty expectations, and described culturally appropriate dress, sarees in particular, as something that increased a sense of dignity and confidence rather than restricting it. This is a useful interpretive frame, but it is offered as one, not as a demonstrated mechanism. The gender-specific attire item (Q18) used different garment prompts for male and female respondents, which means any chi-square association involving that item reflects differing baseline comfort with two different questions, not a clean, isolated gender effect, and the interview data should be read as elaborating on the quantitative pattern rather than explaining its cause.

The regression results point in a similar direction to the correlational findings. Self-Expression confidence was associated with the appearance-related academic-performance belief item (*r* = 0.53) and, once the composite was corrected to remove 9's part-whole overlap, Social Identity confidence showed a comparable, non-inflated association with social media influence (*r* = 0.39). In the regression model, both social influence confidence (β = 0.344, *p* < 0.001) and the appearance-related performance belief (β = 0.379, *p* < 0.001) out predicted gender (β = 0.113, *p* = 0.013) once entered, and gender's coefficient dropped once these variables were added, which is best read as the demographic effect being partially accounted for by psychological variables that overlap with it, not as evidence that gender's influence is transmitted causally through them; a formal mediation test would be required to support that stronger claim, and was not conducted here.

Common method bias is a reasonable concern in a design that collected everything through one self-report questionnaire, but Harman's single-factor test suggests it is not the dominant force behind these associations: a single unrotated factor explained 40.25% of the total variance, comfortably under the 50% threshold usually taken as a warning sign. This does not rule out shared-method variance entirely, but it makes it unlikely to be doing most of the work in the correlations reported above.

The qualitative themes add texture that the survey items could not capture on their own, and the clearest way to describe how they relate to the quantitative results is in terms of convergence, complementarity, and, in one case, productive dissonance, rather than one dataset explaining the other. The armor theme converges with the Self-Expression factor: students describing formal attire as something that helped them feel prepared and less self-conscious mirrors, in their own words, exactly what that factor measures numerically. The social gaze and identity negotiation themes complement the Social Identity factor rather than simply restating it, adding detail the scale items could not, participants talked about specific audiences (classmates, family, strangers on campus) and specific stakes (being judged, being included) that a three-item scale necessarily flattens into a single score. This complementary detail is also a plausible reason that factor showed weaker psychometric performance (ω = 0.61, AVE = 0.34): if social confidence genuinely depends on which audience a student has in mind, a single unidimensional set of items may be struggling to capture something that is not actually unidimensional in students lived experience. The inner self-reliance theme is the clearest case of dissonance. A minority of participants described clothing as largely irrelevant to how confident they felt, which does not contradict the quantitative findings so much as mark their boundary: the average relationships reported here do not hold for everyone, and this theme is a genuine qualification of the pattern rather than an outlier to explain away.

### Theoretical contribution

The Introduction raised a specific theoretical question rather than a geographic one: whether the internal pathway described by enclothed cognition and the external pathway described by impression management remain separable processes when the self is culturally construed as interdependent rather than independent (Markus and Kitayama, 1991). The results speak to this fairly directly. The two factors correlated highly (*r* = 0.79), oblique EFA produced a similarly substantial factor correlation (ϕ = 0.512), and a second-order CFA model, in which a single higher-order factor sat above the two first-order factors, produced statistically identical fit to the freely correlated two-factor model (χ^2^ = 72.41 in both). With only two first-order factors, that equivalence is a mathematical property of the model, not proof that a single construct is the better description, so the finding cannot be read as a verdict on its own. What it does show is that the data are fully consistent with the possibility that these two theorized pathways are less separable here than dual-process accounts built on independent-self samples would predict, and that possibility deserves a real explanation rather than a shrug.

Riemer and Shavitt (2011) offer one. Their experimental work found that impression management operates as a relatively automatic, low-effort process for people from collectivist cultural backgrounds, in contrast with individualists, for whom it is a more deliberate, cognitively effortful one. If presenting oneself appropriately to others is not a separate controlled step for someone with an interdependent self-construal, but something closer to a default mode of self-regulation, then it becomes much less surprising that a measure of internal readiness and a measure of social-evaluation confidence would move together so strongly in this sample. The two constructs may not be merging by accident. They may be reflecting a genuine feature of how confidence is organized for people whose sense of self is, per Markus and Kitayama (1991), constitutively relational rather than privately formed and then presented. Symbolic interactionist accounts of identity point toward a compatible conclusion from different starting assumptions, treating the self as built through interaction rather than expressed after the fact (Ambás and Gárgoles, 2025), which gives this interpretation more than one theoretical lineage behind it.

This reframes what the weaker Social Identity factor might mean. Rather than treating ω = 0.61 purely as a measurement weakness to be fixed in a future revision of the scale, it is worth taking seriously as a possible signal that “social identity confidence” is not one thing for these participants, but several audience-specific and context-specific experiences that a short unidimensional scale compresses into a single number, a possibility the qualitative themes (particularly the social gaze and identity negotiation themes) support directly.

It is worth being honest about how far this argument should be pushed. Enclothed cognition itself has an uneven replication record. A recent z-curve and meta-analytic reassessment found that earlier, widely cited demonstrations show weaker evidential value than more recent studies, meaning the underlying internal mechanism this study builds on is still being actively tested and revised in its country of origin, let alone once transplanted into a new cultural setting (Horton et al., 2025). The present findings should therefore be read as culturally situated evidence, consistent with a self-construal account of why internal and external confidence pathways converge in this sample, rather than as a settled demonstration that they always do in collectivist contexts generally. What can be said with more confidence is narrower and still useful: applying enclothed cognition and impression management theory to a Bangladeshi sample without adapting for self-construal would likely have missed this convergence entirely, and that is the theoretical contribution this study is best positioned to make.

### Implication

Theoretically, this study suggests that dual-process models separating internal and external routes to appearance-related confidence may need cultural qualification rather than universal application. Future cross-cultural work could treat the degree of separation between these pathways as a variable to be measured, using self-construal as a candidate explanatory factor, rather than assuming a fixed two-factor structure travel unchanged across cultural settings.

The practical implications are broader than dress policy alone, and they are offered here as considerations for relevant stakeholders and starting points for future intervention research, not as settled recommendations, since a cross-sectional design cannot establish that changing clothing would change confidence. For higher education more broadly, the finding that students associate certain attire with a felt sense of readiness suggests institutions might think about how dress codes, or the absence of them, interact with student comfort and confidence, particularly around high-stakes moments like presentations and interviews. For student wellbeing and counseling services, the qualitative theme of inner self-reliance is a useful caution against treating appearance-focused interventions as a default; some students explicitly locate their confidence elsewhere, and support services working with appearance-related anxiety might screen for this rather than assume clothing is always part of the picture. For career preparation, the strength of the appearance-related academic-performance belief in the regression results suggests students may benefit from explicit conversations about interview attire and professional presentation, delivered as one tool among several rather than the primary lever. For organizational psychology, the pattern of internal and external confidence pathways running closely together in this collectivist sample raises a genuine question about whether workplace dress norms function differently for interdependent-self employees than the largely Western literature on professional attire assumes. For fashion education and design programs, the qualitative emphasis on comfort, authenticity, and culturally meaningful garments like the saree and panjabi suggests curricula could usefully engage students in thinking about clothing as psychological and cultural infrastructure, not just aesthetic choice.

Across all of these, the same caveat applies. What this study shows is association, in a specific cultural setting, at a single point in time. Turning any of the directions above into an actual intervention or policy would need its own dedication, ideally longitudinal or experimental, research design before firmer claims are warranted.

### Limitation

Several limitations should be acknowledged. First, cross-sectional design prevents causal conclusions, and longitudinal or experimental research is needed to examine causal pathways more directly. Second, the sample was drawn primarily from public universities and was gender imbalanced (approximately 72% male), limiting generalizability. Further limitation concerns the attire-confidence item (Q18), which used gender-specific garment prompts rather than an identical item across groups; future research should use a common, gender-neutral formulation to allow a cleaner comparison.

Measurement invariance across gender was tested but could not be fully established, as configural fit was adequate for male respondents but fell short of conventional thresholds for the smaller female subsample (*n* = 85). Reported gender differences therefore reflect observed-score, not latent-score, comparisons; replication with a larger, more gender-balanced sample is needed to confirm measurement equivalence across groups.

The factor structure itself warrants some caution. Although the two-factor model was supported by confirmatory fit indices and discriminant validity testing (HTMT = 0.696), parallel analysis suggested a single-factor solution may also be viable, and a second-order model produced statistically identical fit to the two-factor model, a property of having only two first-order factors rather than independent evidence favoring either structure. Taken together, these results support retaining the two-factor model while acknowledging that the two constructs remain closely related and would benefit from a larger, more balanced item pool in future work. Relatedly, the Social Identity construct demonstrated weaker psychometric performance (α ≈0.59; ω = 0.61; CR = 0.60; AVE = 0.34), possibly reflecting conceptual breadth and limited item coverage, and should continue to be interpreted as exploratory.

Finally, because all variables were collected through self-report on a single questionnaire, common-method bias and social-desirability effects cannot be fully ruled out despite qualitative triangulation. Harman's single-factor test offered some reassurance here, a single unrotated factor accounted for 40.25% of total variance, below the 50% threshold conventionally associated with substantial bias, though this test alone does not eliminate the concern entirely. Content validity was supported by expert and pilot feedback but not established through a formal procedure such as a Content Validity Index; future work should include structured expert-panel ratings and cognitive interviewing prior to broader use of this instrument. Cultural specificity may also limit applicability beyond this setting, highlighting the need for replication in different populations and cultural contexts.

## Future research

Future research should strengthen measurement development by refining the Social Identity dimension and incorporating additional culturally relevant indicators, such as peer influence, family expectations, institutional norms, and social media pressure, given the qualitative evidence that social confidence may be more audience-specific than a short unidimensional scale can capture. A larger, more gender-balanced sample would allow measurement invariance to be more fully established across groups, building on the configural analysis conducted here, which was limited by the smaller female subsample. Relatedly, whether the two-factor structure is best treated as two related but distinct constructs, or as closely overlapping facets of a broader clothing-confidence construct, remains an open question that a larger item pool and a larger sample could help settle more decisively than the present study's second-order comparison could.

Because the present interpretation rests on self-construal as a plausible explanation for why internal and external confidence pathways converged so strongly in this sample, future work would benefit from measuring self-construal directly, using an established scale, rather than inferring it from national or cultural context alone. This would allow a more direct test of whether individual differences in independent vs. interdependent self-construal, rather than culture as a broad proxy, account for the strength of this relationship, and would also make cross-cultural comparison with individualist samples more precise. Experimental and longitudinal designs remain necessary to clarify causal direction and temporal sequence between clothing and confidence, and future studies could usefully incorporate objective performance measures alongside the perception-based items used here. Finally, culturally sensitive interventions and research into how social media, fast fashion, and digital self-presentation shape clothing-related confidence among young adults would extend this work into territory the present design could not address.

## Conclusion

This mixed-methods study suggests that clothing plays a meaningful role in shaping self-confidence among Bangladeshi university students, best understood through culturally situated psychological processes rather than enclothed cognition or impression management theory applied unchanged from individualist settings. A provisional, internally cross-validated two-factor structure distinguished Self-Expression and Performance Confidence from Social Identity and External Influence Confidence, both associated with self-expression confidence beyond demographic variables alone, an association rather than a demonstrated causal pathway.

The strong relationship between the two factors (*r* = 0.79), and their statistically equivalent fit under a second-order model, is consistent with self-construal theory's prediction that internal and external routes to confidence are less separable for people with interdependent rather than independent self-construal, a pattern for which prior cross-cultural work on impression management offers a plausible, though not yet confirmed, mechanism. Harman's single-factor test suggested common method bias was unlikely to be the dominant explanation for these associations.

Self-Expression showed solid psychometric support, while Social Identity remained comparatively weak and should continue to be treated as exploratory; the qualitative finding that social confidence depended on which audience students had in mind suggests this weakness may reflect genuine construct complexity rather than measurement failure alone.

Overall, the findings extend enclothed cognition and impression management theory to a collectivist South Asian context by treating their assumed internal/external separation as an empirical question rather than a given, and offer directions, not validated recommendations, for higher education, student counseling, career preparation, and fashion education. The results support a culturally attentive understanding of clothing's psychological role, one that takes its potential seriously without overstating what a cross-sectional, self-report design can demonstrate.

## Data Availability

The raw data supporting the conclusions of this article will be made available by the authors, without undue reservation.
